# CUG-BP1 regulates RyR1 ASI alternative splicing in skeletal muscle atrophy

**DOI:** 10.1038/srep16083

**Published:** 2015-11-04

**Authors:** Yinglong Tang, Huiwen Wang, Bin Wei, Yuting Guo, Lei Gu, Zhiguang Yang, Qing Zhang, Yanyun Wu, Qi Yuan, Gang Zhao, Guangju Ji

**Affiliations:** 1 National Laboratory of Biomacromolecules, Institute of Biophysics, Chinese Academy of Sciences, 15 Datun Road, Beijing 100101, China; 2 Department of Physiology, Wayne State University School of Medicine, Detroit, Michigan 48201; 3 University of the Chinese Academy of Sciences, Beijing 100049, China; 4 Department of Neurology, Xijing Hospital, Forth Military Medical University, Xi’an, Shaanxi 710032, China; 5 Department of Anesthesiology, Beijing Tiantan Hospital, Capital Medical University, Beijing 100050, China

## Abstract

RNA binding protein is identified as an important mediator of aberrant alternative splicing in muscle atrophy. The altered splicing of calcium channels, such as ryanodine receptors (RyRs), plays an important role in impaired excitation-contraction (E-C) coupling in muscle atrophy; however, the regulatory mechanisms of ryanodine receptor 1 (RyR1) alternative splicing leading to skeletal muscle atrophy remains to be investigated. In this study we demonstrated that CUG binding protein 1 (CUG-BP1) was up-regulated and the alternative splicing of RyR1 ASI (exon70) was aberrant during the process of neurogenic muscle atrophy both in human patients and mouse models. The gain and loss of function experiments *in vivo* demonstrated that altered splicing pattern of RyR1 ASI was directly mediated by an up-regulated CUG-BP1 function. Furthermore, we found that CUG-BP1 affected the calcium release activity in single myofibers and the extent of atrophy was significantly reduced upon gene silencing of CUG-BP1 in atrophic muscle. These findings improve our understanding of calcium signaling related biological function of CUG-BP1 in muscle atrophy. Thus, we provide an intriguing perspective of involvement of mis-regulated RyR1 splicing in muscular disease.

Muscle atrophy is featured by rapid loss of muscle mass and contractile function, which results from fasting, denervation, disuse as well as a co-morbidity of several common diseases, including cancer, sepsis, uremia, and diabetes^1^,^2^. In general, muscle atrophy involves several pathways of intracellular signaling that eventually activate the protein degradation mechanisms including ubiquitin-proteasome and autophagy-lysosome system, which work coordinately to remove the myofibrillar proteins and myocyte organelles^3^,^4^. The related E3 ligase genes, Atrogin-1/MAFbx and MuRF-1, as well as several autophagy genes, including LC3, Bnip-3, have been found to be rapidly up-regulated at early stage of multiple types of muscle atrophy to initiate protein degradation^5^,^6^,^7^,^8^.

Post-transcriptional regulation of RNA processing plays an essential role in gene expression during mammalian development^9^ as well as pathogenesis^1^,^2^. Alternative splicing is a process in which multiple messenger RNAs (mRNAs) are generated from one pre-messenger RNA (pre-mRNA) molecule, and as a result, distinct proteins isoforms, or splicing variants, are produced with different biological functions^10^. Given that 92–94% of human genes undergo alternative splicing^11^,^12^, alternative splicing provides an essential mechanism for gene expression and regulation. An increasing number of studies has shown that disruption of normal splicing patterns can cause or modify human disease^13^,^14^; however, the mechanisms by which aberrant alternative splicing play a role in muscular human disease are still unclear.

A large family of RNA-binding proteins (RBPs) as splicing regulators has been identified with involvement in multiple regulatory functions in mammalian cells. The CUG-BP1 and ETR-3 like factor (CELF) family consists six members that control various aspects of RNA metabolism, including alternative splicing, mRNA stability and translation^15^,^16^. The majority of studies on CUG-BP1/CELF1 has been focused on the roles of this protein in development of muscle pathology in myotonic dystrophy (DM) type 1 and 2 patients^17^,^18^. Dysfunctional calcium channel genes with alternatively spliced exons have been identified in human DM1 disease, especially RyR1 ASI (exon70, residues 3481–3485)^19^. Some other genetic disorders are also suggested to be partially caused by the mutation of RyR1 ASI region^20^. Meanwhile, Ca^2+^ dependent process known as excitation-contraction (E-C) coupling is also mis-regulated in atrophy related diseases^21^,^22^ and the ASI region has been found to be involved in regulating efficacy of skeletal muscle E-C coupling *in vitro*^21^,^23^. However, whether the aberrant alternative splicing of calcium regulatory genes exist in neurogenic muscle atrophy and the regulatory mechanism remains unknown.

In this study, we examined the biological function of CUG-BP1 and altered RyR1 alternative splicing in both human neurogenic muscle atrophy and denervation-induced muscle atrophy mouse model. We found that CUG-BP1 up-regulation led to aberrant alternative splicing of RyR1 and impaired Ca^2+^ release in muscle atrophy. Most interestingly, by silencing CUG-BP1 in denervated muscle, we identified altered calcium release properties, as well as the extent of atrophy was significantly prevented. These findings suggest that an altered function of CUG-BP1 plays a pivotal role in the pathogenic process of skeletal muscle atrophy, and CUG-BP1 can be served as a target for clinical intervention of neurogenic skeletal muscle atrophy.

## Results

### Up-regulation of CUG-BP1 and aberrant splicing of RyR1 ASI in skeletal muscle atrophy

**Figure 1:**
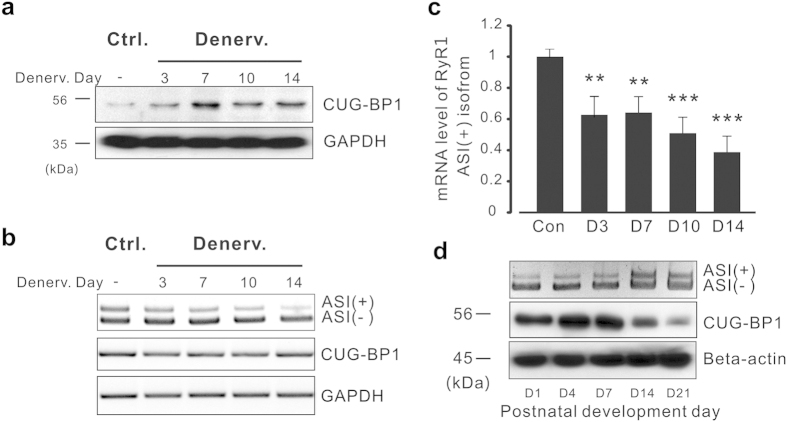
Increased CUG-BP1 protein level and decreased ASI(+) isoform in denervation-induced skeletal muscle atrophy of mice. (**a**) Western blotting results show CUG-BP1 protein level is increased in the process of denervation-induced atrophy in TA muscle. (**b**) mRNA expression level of CUG-BP1 and RyR1 ASI alternative splicing determined by RT-PCR in the process of denervation-induced atrophy in TA muscle. (**c**) Real time PCR analysis of RyR1 ASI(+) proportion in 3, 7, 10, 14 days after denervation. (**d**) The correlation between the increased RyR1 ASI(+) isoform and the developmental loss of CUB-BP1 protein during three weeks after postnatal. To keeping the clarity and conciseness, the cropped gels and blots were presented in the current and following figures. The full-length gels/blots are included in the supplementary information. Three independent experiments were performed and one representative result was presented. Data are presented as the means ± s.e.m. **P < 0.01 and ***P < 0.001 by two-tailed Student’s test.

It has been reported that CUG-BP1, a protein interacts with RNA CUG repeats, is expanded in patients with DM1^24^,^25^, and the activation of CUG-BP1 leads to dysfunction in splicing, stability and translation of multiple mRNAs^26^. To investigate the role of CUG-BP1 in neurogenic muscle atrophy, we established neurogenic skeletal muscle atrophy model by sciatic denervation on hind limb muscles from three-month-old mice. As shown in Fig. 1a, western blotting assay indicated that the protein level of CUG-BP1 was up-regulated in denervated tibialis anterior (TA) muscle at day 3 and reached the plateau at day 7 after the denervation; however, reverse transcription polymerase chain reaction (RT-PCR) assay indicated that the mRNA level of CUG-BP1 was not altered by denervation (Fig. 1b), which suggested that the increase in CUG-BP1 protein level was attributed to the increase of post-transcription efficiency.

In order to investigate the splicing isoforms of RyR1 ASI during muscle atrophy, RT-PCR analysis was performed with a primer set generated fragments of different length, according to the inclusion and the exclusion of the exon70 of RyR1. The proportion of variant ASI(+), which including ASI region, was significantly decreased in the following two weeks after denervation (Fig. 1b). To quantify this process, quantitative PCR (qPCR) was performed with specific primers targeted to RyR1 ASI(+) isofrom, the results showed that ASI(+) variant was down-regulated to 62.7% of the contralateral in TA muscle at day 3 and eventually decreased to 38.7% at day 14 after denervation (Fig. 1c). The result revealed the same pattern of RyR1 aberrant splicing as Fig. 1B. However, the mRNA expression level of RyR1 gene was not significantly changed in the atrophic TA muscle, suggesting that alternative splicing, but not the gene expression activity, plays an important role in RyR1 pathological remodeling (see Supplementary Fig. S1a) in skeletal muscle atrophy.

The results above led us to further investigate the potential involvement of CUG-BP1 in RyR1 alternative splicing during postnatal development in physiological condition. We examined the expression of CUG-BP1 and RyR1 ASI in skeletal muscles at different postnatal stages of mice. Western blotting assay revealed that the amount of CUG-BP1 expression reached to peak level on postnatal day 1 in TA muscle (Fig. 1d), in the following three weeks, the protein level of CUG-BP1 was continuously down-regulated; interestingly, the proportion of RyR1 ASI(+) was persistently increased in the same process, suggesting the quantitative role played by CUG-BP1 in determining physiological status of RyR1 alternative splicing.

**Figure 2:**
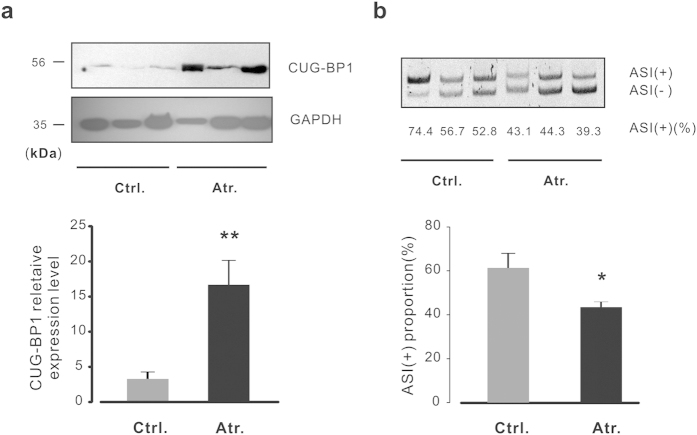
The changes of CUG-BP1 expression and alternative splicing of RyR1 in human neurogenic atrophy. (**a**) Protein level of CUG-BP1 in control and neurogenic atrophic human skeletal muscle was detected by western blot assay (upper panel), the statistic results of densitometry analysis showed that the expression of CUG-BP1 was enriched in atrophic human skeletal muscle (lower panel). (**b**) Analysis of RyR ASI splicing pattern was performed by RT-PCR and then separated on an 8% polyacrylamide gel, the statistic results showed the proportion of RyR1 ASI(+) isoform was decreased accordingly (lower panel). Data are presented as the means ± s.e.m from three control and three atrophic human gastrocnemius muscle samples; *P < 0.05 and **P < 0.01 by two tailed Student’s test.

To further elucidate the role of CUG-BP1 in human muscle diseases, western blot analysis was performed in control and neurogenic atrophic human gastrocnemius muscles. As shown in Fig. 2a (upper panel), the protein level of CUG-BP1 was significantly increased in neurogenic muscle atrophy. The alternative splicing study of RyR1 demonstrated that the variant ASI(+) decreased in the atrophic group (Fig. 2b, right panel). Interestingly, patients with higher degree of increased CUG-BP1 protein level showed a lower degree of ASI(+) proportion, suggesting a significant correlation exists between CUG-BP1 and RyR1 ASI pattern.

### Correlation between alternative splicing of RyR1 ASI and CUG-BP1 protein level in different muscles

**Figure 3:**
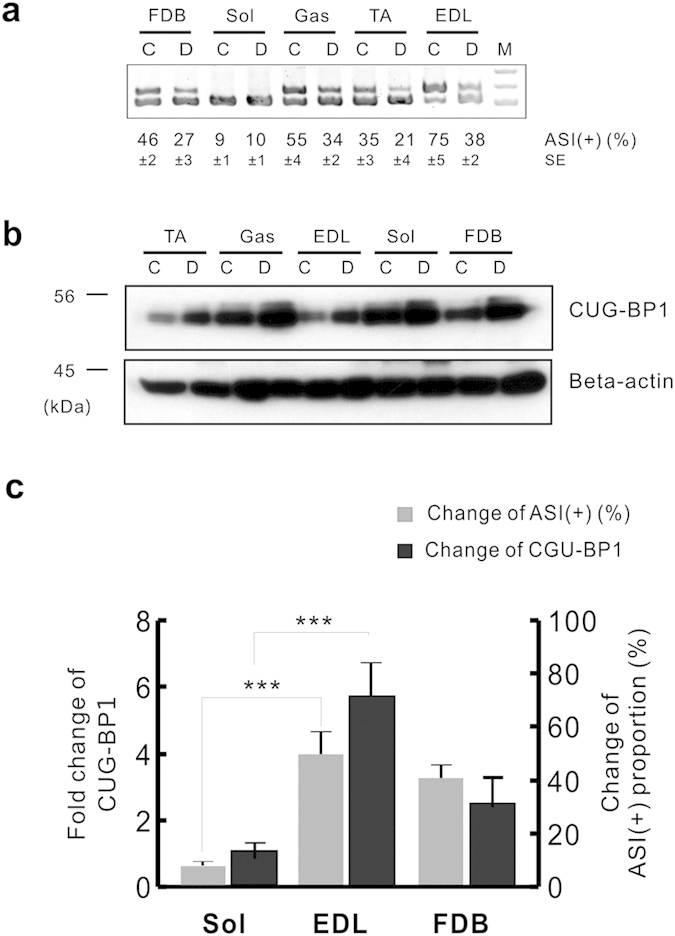
Positive correlation between alternative splicing of RyR1 ASI and CUG-BP1 protein level. (**a**) The proportion of ASI(+) in atrophy muscle in different types of denervated and contralateral striated muscles from three-month-old adult wild type mice. PCR bands were quantified by densitometry. (**b**) Western blot analysis of CUG-BP1 expressions in denervated and contralateral muscles. Western blots were quantified by densitometry and normalized to β-actin. (**c**) The positive correlation of changes in CUG-BP1 expression and ASI(+) proportion in denervated muscles. Fold changes of CUG-BP1 protein level compared to contralateral muscles were shown. At least three independent experiments were performed and one of the results was presented. Data are presented as the means ± s.e.m. ***P < 0.001 by two-tailed Student’s test.

To verify whether CUG-BP1 provided a general mechanism in mediating alternative splicing of RyR1 ASI in neurogenic muscle atrophy, splicing patterns of RyR1 ASI was examined in samples collected from soleus (Sol), gastrocnemius (Gas), TA, extensor digitorum longus (EDL), and flexor digitorum brevis (FDB) muscles. As shown in Fig. 3a, the proportion of ASI(+) varied in different types of muscle. Being a slow twitch-dominant muscle, soleus showed the lowest ASI(+) (9 ± 1%) level; while EDL, a typical fast twitch muscle, exhibited the highest ASI( + ) (75 ± 5%) level. Interestingly, CUG-BP1 was highly expressed in soleus, but much lower in EDL muscle (Fig. 3b). These results suggest that the proportion of RyR1 ASI(+) isoform is negatively correlated to the protein level of CUG-BP1 in physiological conditions. Next, we examined the change of CUG-BP1 expression and RyR1 ASI(+) proportion in denervation-induced muscle atrophy. As a typical fast twitch muscle, EDL exhibited the highest reduction of ASI(+) proportion from 75 ± 5% to 38 ± 2%, while soleus remained expressed the ASI(+) isoform dominantly (10 ± 1%) (Fig. 3c). This was consistent with the findings that CUG-BP1 was up-regulated by 5.71 ± 0.99 folds in EDL muscle, but only 0.65 ± 0.32 folds in soleus muscle which with highly expressed CUG-BP1. Competitively, FDB, a moderate fast twitch muscle^27^, exhibited a 2.33 ± 0.63 folds increase in CUG-BP1 protein level and 41 ± 3% of ASI(+) decrease after denervation (Fig. 3c). All data above suggests that CUG-BP1 mediates RyR1 ASI alternative splicing in the process of muscle atrophy.

**Figure 4:**
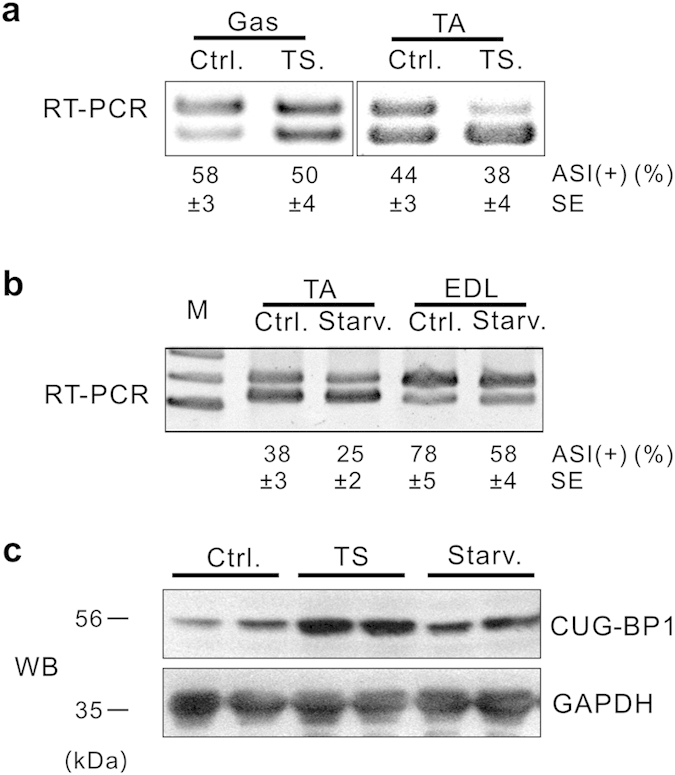
Alteration of CUG-BP1 and ASI proportion in TS and Star induced muscle atrophy. (**a**) Proportion of ASI(+) in Gas and TA muscles from tail suspension (TS) mice was analyzed by RT-PCR. (**b**) Proportion of ASI(+) in TA and EDL muscles from starvation (Starv) mouse model was determinated by RT-PCR analysis. (**c**) Increased CUG-BP1 expression was confirmed by WB assay in TA muscle from TS or Starv mice. Data are presented as the means ± s.e.m.

To determine whether the increases in CUG-BP1 protein level and altered ASI splicing are common features in different types of muscle atrophy, we next examined muscle atrophy induced by tail suspension (TS) and starvation (Starv), which are commonly used models in muscle atrophy studies^28^,^29^. As shown in Fig. 4, inclusion of ASI was remarkably decreased (Fig. 4a,b) with the concurrent increase in the CUG-BP1 protein level (Fig. 4c). Thus, the increases in CUG-BP1 protein level and alteration of ASI splicing are conjoint phenomenon in several types of muscle atrophy.

### Subcellular distribution of CUG-BP1 during the process of muscle atrophy

**Figure 5:**
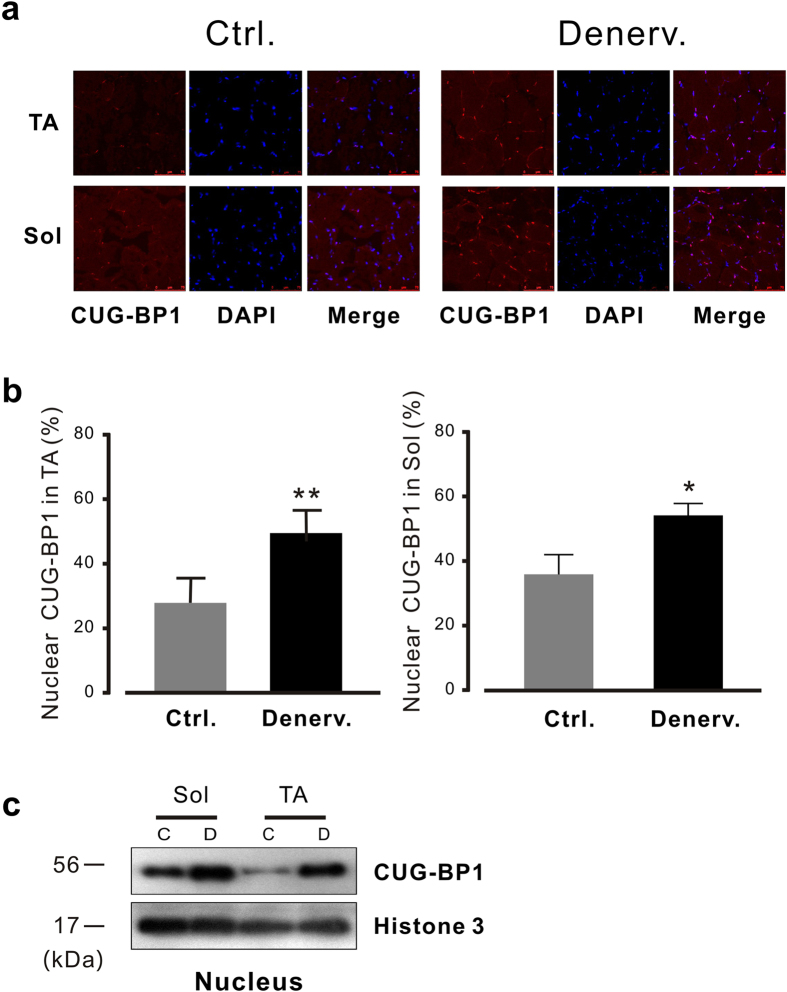
Subcellular distribution of CUG-BP1 in atrophied muscles. (**a**) Immunofluorescence staining of CUG-BP1 from control and denervated muscle by frozen section. The localization of CUG-BP1 was determined by immunofluorescence staining. DAPI and CUG-BP1 (Alexa Fluor® 546)-stained images of a single representative field are shown and merged. (**b**) Summary data of Subcellular distribution of CUG-BP1. Notably, at day 7 after denervation, total muscle nuclei stained with CUG-BP1 was increased in TA (left) and in soleus muscl (right). (**c**) The results of western blots from subcellular fractions of muscles after denervation confirm the increase of CUG-BP1 expression in nucleus. Nuclear extracts were prepared from denervated and control TA or soleus muscle. Relative amounts of CUG-BP1 during atrophy as well as the nuclear marker Histone 3 were determined by Western blotting. Data are presented as the means ± s.e.m; *P < 0.05 and ** P < 0.01 by two-tailed Student’s test.

CUG-BP1 has been identified a critical player in regulating mRNA stability in cytoplasmic and alternative splicing in nucleus^15^,^16^. The subcellular location and activity of CUG-BP1 are precisely determined by multiple post-translational modification mechanisms^30^,^31^. To determine whether the subcellular translocation of CUG-BP1 is involved in regulation of CUG-BP1 function during muscle atrophy, we performed immunofluorescence staining assay. We observed that CUG-BP1 was predominant in nucleus and the nuclear presence of CUG-BP1 was strikingly enhanced in TA and soleus upon denervation(Fig. 5a). Western blots from nuclear fractions of TA or Gas muscles after denervation for 7 days also confirmed the increase in CUG-BP1 expression in nucleus (Fig. 5b, right). The enhanced nuclear presence of CUG-BP1 in atrophy muscle is consistent with its function in regulating alternative splicing of RyR1^32^.

### CUG-BP1 directly regulates RyR1 ASI alternative splicing

**Figure 6:**
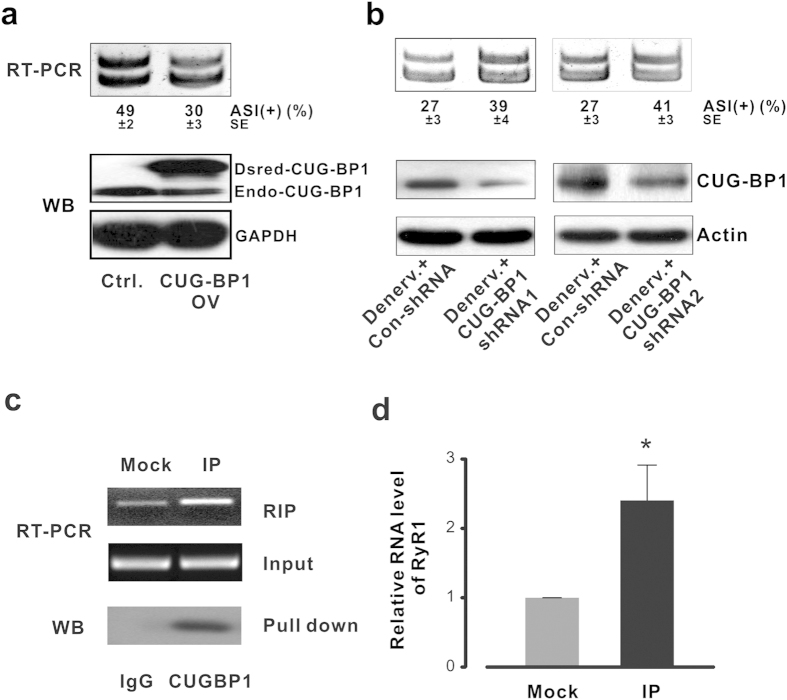
RyR1 ASI alternative splicing is directly regulated by CUG-BP1. (**a**) Dsred tagged mouse CUG-BP1 cDNA was over-expressed in FDB muscle. Ten days after electroporation, over-expression of CUG-BP1 was verified by western blot. The proportion of RyR1 ASI(+) isform was decreased from 49 ± 2% to 30 ± 3%. (**b**) CUG-BP1 knock-down assay in denervated FDB. The protein level of CUG-BP1 was inhibited in both shRNA assays; RyR1 ASI(+) isoform was increased from 27 ± 3% to 39 ± 4% in shRNA 1 and 41 ± 3% in shRNA 2 samples. (**c**) RIP analysis in undifferentiated C2C12 cells. IP was performed using antibodies against CUG-BP1, and RT-PCR assay using primers targeting adjacent intron of RyR1 exon 70 as indicated. Beta-actin was used as input control. (**d**) Real time PCR assay using the same primers targeting adjacent intron of RyR1 exon 70. Data are presented as the means ± s.e.m.; *P < 0.05.

In order to examine whether the upregulated CUG-BP1 function is sufficient to cause aberrant splicing of RyR ASI, as observed in muscle atrophy, and to determine the internal correlation between RyR1 ASI splicing and CUG-BP1 expression, we performed expressional manipulation of CUG-BP1 using the *in vivo* electroporation^33^ followed by RT-PCR and western blot (WB) analysis. None persistent injury and inflammation response of the muscles were observed 5 days post plasmids injection. In FDB muscle, the pre-mRNA of the RyR1 was spliced to a proportion of 49% ASI(+) when transfected with control plasmid. However, when the FDB muscle was transfected with the CUG-BP1 expression plasmid, ASI(+) proportion was decreased from 49 ± 2% to 30 ± 3% (Fig. 6a, top). Over-expression of CUG-BP1 resulted in the atrophy-like RyR1 ASI splicing pattern though the total mRNA level of RyR1 remained unchanged (see Supplementary Fig. S1b).

Moreover, in order to further determine whether the aberrant slicing of RyR1 ASI was caused by up-regulation of CUG-BP1 function, we generated two shRNA plasmids that target CUG-BP1 coding sequence. The knockdown of CUG-BP1 was performed by using the *in vivo* electroporation in FDB muscle^33^. Ten days after electroporation, FDB muscles were collected and followed by RT-PCR and WB analysis. We found that the expression level of endogenous CUG-BP1 protein in denervated FDB muscle was significantly depressed after transfected with CUG-BP1 shRNA (Fig. 6b). Correspondingly, when the denervated FDB muscle was transfected with the shRNA, RyR1 ASI(+) proportion was increased from 27 ± 3% to 39 ± 4% (shRNA-1) and 41 ± 3% (shRNA-2), respectively (Fig. 6b), indicating that shRNA-mediated down-regulation of CUG-BP1, at least partially, rescued aberrant ASI splicing during FDB muscle atrophy.

We next explored whether CUG-BP1 directly regulated RyR1 ASI pre-mRNA splicing in muscle atrophy using a mouse myoblast cell line C2C12. The physical association of CUG-BP1 and RyR1 was examined by RNA immunoprecipitation (RIP) PCR assay. The coprecipitated RNA was subjected to RT-PCR to demonstrate the presence of RyR1 pre-mRNA (Fig. 6c). As an independent validation of the association with CUG-BP1, RIP-quantitative PCR assay was performed for these transcripts (Fig. 6d). Our results confirm that CUG-BP1 specially bond to RyR1 pre-mRNA by using β-actin as a negative control.

### CUG-BP1 alters calcium release properties in FDB myofibers

**Figure 7:**
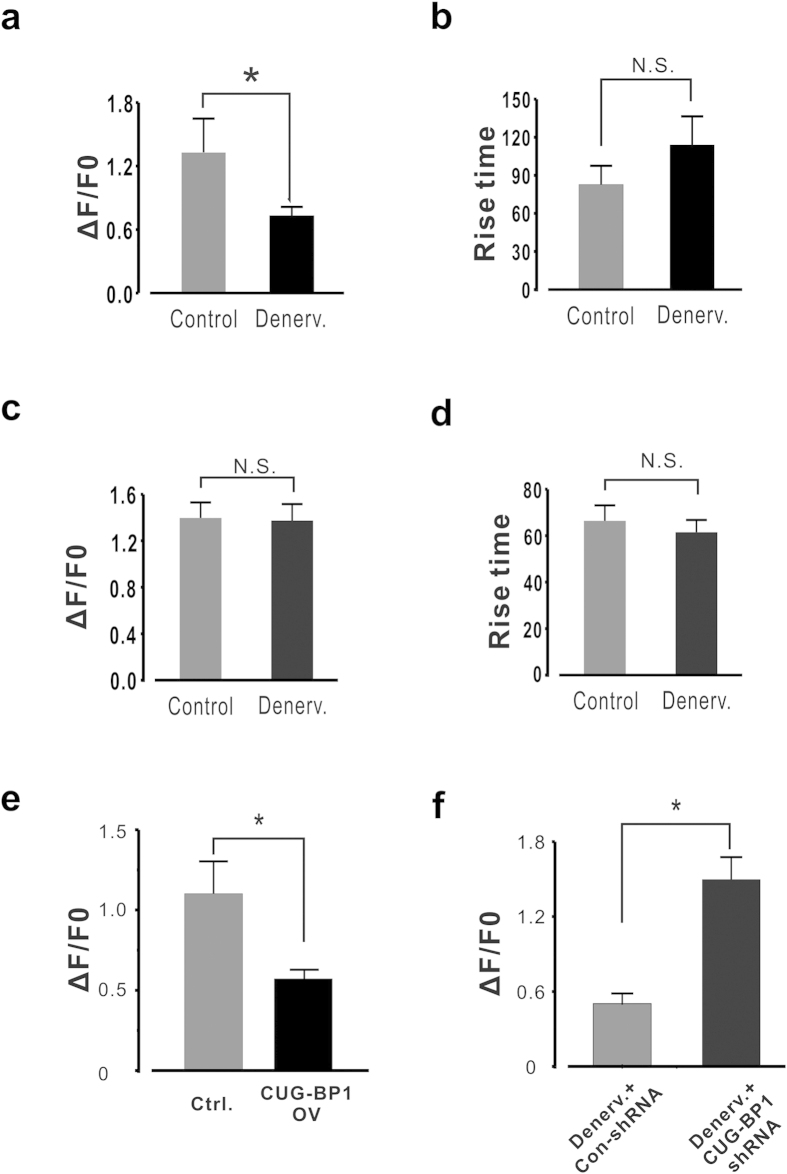
CUG-BP1 expressional manipulation alters Ca2 + release properties in FDB muscle fibers. (**a,b**) Summary data of Ca^2+^ release properties in control and atrophic FDB muscle fibers. Peak amplitude of 1 mM caffeine induced Ca^2+^ transient was significantly lower in atrophic myofiber than that of control. Rise time of Ca^2+^ transient in in atrophic myofiber was significantly prolonged. n = 15 ~ 20 from 6 mice. (**c,d**) Summary data of Ca^2+^ content measured by 10 mM caffeine in control and atrophic FDB muscle fibers. (**e**) Ca^2+^ release properties induced by 1 mM caffeine in control and CUG-BP1 over-expressed FDB muscle fibers. Peak amplitude of Ca^2+^ transient was significantly lower in CUG-BP1 over-expressed myofiber than that of control. n = 13 from 5 mice. (**f**) Peak amplitude of Ca^2+^ transient in denervated FDB myofiber with CUG-BP1 shRNA. It is notable that knocking down CUG-BP1 in atrophic process dramatically increased the peak amplitude of Ca^2+^ transient. n = 17 from 7 mice. Data are presented as the means ± s.e.m.; * P < 0.05 by two-tailed Student’s test.

The contribution of aberrant endoplasmic reticulum (ER) Ca^2+^ release has been recognized as an important pathogenic factor of muscular dystrophy^34^. In this study we tested whether the CUG-BP1 aberrant expression leads to any observable change in sarcoplasmic reticulum (SR) Ca^2+^ release property in intact skeletal myocytes. Single FDB myofibers were separated from contralateral and denervated (Day 7) muscles, and caffeine-induced Ca^2+^ release was characterized. The peak amplitude of Ca^2+^ release in the denervated myofibers was lower than that of the control myofibers when 1mM caffeine was applied (Fig. 7a); the rise time was also prolonged by 68.5% in the atrophic cells (Fig. 7b), which again characterized the desynchronized E-C coupling in muscle atrophy. However, there was no difference of SR Ca^2+^ content between control and denervated myofibers (Fig. 7c,d). Previous studies showed that the open probability of ASI(-) RyR1 in planar lipid bilayers is less than that of ASI(+)^19^, therefore these findings are quite consistent with the RyR1 channel isoform changes from ASI(+) to ASI(-), and suggested an aberrant calcium release function of the RyR1 ASI (+) in muscle atrophy.

Similar experiments were performed in control and CUG-BP1 overexpressing FDB muscles. We found that the peak amplitude of Ca^2+^ release in the CUG-BP1 overexpressing myofibers was much lower than that of the control, exhibiting the same pattern as the atrophic myofibers (Fig. 7e). Furthermore, when denervated myofibers were transfected with CUG-BP1 shRNA, the peak amplitude of caffeine-induced Ca^2+^ release was increased to a much higher level than the atrophic myofibers (Fig. 7f). Taken together, our results suggest that up-regulated CUG-BP1 alters calcium release properties in FDB myofibers. The causal relationship between CUG-BP1 altered calcium release properties and RyR1 alternative splicing will be studied in the future.

### Gene silencing of CUG-BP1 by shRNA protects FDB muscle from atrophy

**Figure 8:**
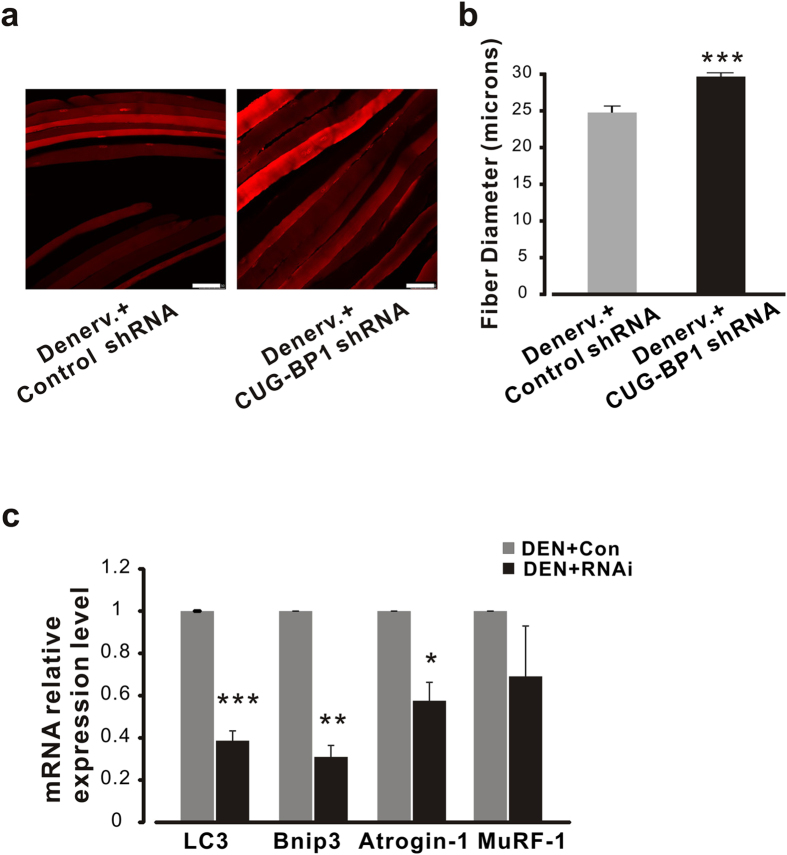
CUG-BP1 knockdown protects denervation FDB muscle from atrophy. (**a**) Representative images of FDB muscle fibers transfected with control and CUG-BP1 shRNA. Images were taken at day 10 after denervation with a total 400 × magnitude. (**b**) Statistic result of FDB myofiber diameters transfected with CUG-BP1 shRNA. n = 120 from 4 mice. (**c**) Relative mRNA levels of muscle atrophy-related genes in shRNA-CUG-BP1 denervated muscles. The mRNA levels of LC3, Bnip3, and Atrogin-1 in denervated shRNA-CUG-BP1 muscles were decreased to 38.7 ± 4.6%, 31.0 ± 5.4% and 57.5 ± 8.7% of those in the control shRNA muscles. Data are presented as the means ± s.e.m. *P < 0.05, **P < 0.01 and ***P < 0.001 by two-tailed Student’s test.

Down-regulation of CUG-BP1 rescues the aberrant Ca^2+^ signaling leads us to hypothesize that CUG-BP1 plays an important role in muscle atrophy. We, thus, further tested the idea whether the pathologic process of muscle atrophy could be intervened through targeting CUG-BP1. To quantify the influence of CUG-BP1 gene silencing on muscle atrophy, we measured and calculated the diameter of atrophy muscle fibers transfected with shRNA plasmid after denervation (Fig. 8a). As shown in Fig. 8b, after 10 days of shRNA injection, the diameter of FDB myofiber was not significantly reduced (maintained at 29.7 ± 0.6μm, comparing to normal size 30.3 ± 1.0μm, data not shown); however, the control denervation myofiber diameter was only 24.8 ± 0.9 μm. These results indicate myofiber atrophy was partially rescued by correction of abnormal CUG-BP1 expression.

The fundamental roles of CUG-BP1 playing in muscle atrophy encouraged us to investigate the expression of the genes functionally involved E3 ligases (Atrogin-1 and MuRF-1) and autophagy (LC3 and Bnip3) mediated protein degradation pathway^3^,^35^. As shown in Fig. 8c, ten days after denervation, the relative expressional levels of LC3 and Binp3 were dramatically decreased in CUG-BP1 shRNA FDB muscle compared to those of the contralateral limb. Moreover, the relative expressional levels of Atrogin-1 and MuRF-1 gene were also decreased in CUG-BP1 shRNA muscles though statistically less dramatic, suggesting that CUG-BP1 mainly exerts its effect through autophagy dependent pathway in neurogenic atrophy.

## Discussion

The biological functions of CUG-BP1 in skeletal muscle diseases, including neurogenic muscle atrophy, are mostly unknown. Although several earlier studies demonstrated that CUG-BP1 is involved in myotonic dystrophy, whether a dys-regulated CUG-BP1 function provides a general mechanism mediating skeletal muscle disease remains unknown. In the present study, we demonstrated for the first time that CUG-BP1 was up-regulated both in neurogenic muscle atrophy patients and muscle atrophy mouse models, suggesting that up-regulation of CUGBP-1 protein level is a common feature during initiation of muscle atrophy. Furthermore, when the CUG-BP1 up-regulation is inhibited by the specific shRNA in denervation-induced muscle atrophy, muscle atrophy process was strikingly prevented, which suggests that CUG-BP1 is a potential therapeutic target in the treatment of skeletal muscle atrophy.

Post-transcriptional regulation of CUG-BP1 has been implicated in the translation and phosphorylation^36^,^37^,^38^. Considering that the up-regulated miR-23 and miR-503 expression in skeletal muscle atrophy^39^,^40^ which would represses CUG-BP1 translation^36^,^37^, translational regulation is not attributed to the increase of CUG-BP1 protein level in the skeletal muscle atrophy. Previous study indicated that the increase in the CUG-BP1 expression level is due to mutant CUG repeats^41^ and PKC-mediated hyperphosphorylation^38^ in DM1. In this study, we did not observe any change of mutant CUG repeats or PKCα, βII expression level in the denervated mice (see Supplementary Fig. S2). However, it has been reported that CUG-BP1 is phosphorylated by cyclinD3-cdk4/6 at Ser302, which enhances CUG-BP1 binding to certain mRNA^30^. Considering the expression level of cyclin D3 and cdk 4 was strikingly increased in the denervation model (see Supplementary Fig. S2), it is possible that cyclinD3-cdk4/6 increases the steady-state levels of CUG-BP1 via phosphorylation. All these results hint a new way of activating CUG-BP1 expression by post-transcriptional regulation in denervation-induced muscle atrophy exists.

Programmatic switch that specifically reverts alternative splicing regulation to an embryonic state is important in many diseases^13^. A recent study reported that CUG-BP1 regulates re-expression of embryonic M2 isoform of pyruvate kinase that correlates with altered glucose metabolism in DM1^42^. The ASI residues are lacking in the fetal RyR1 isoform (ASI(-) RyR1), but present in the adult isoform (ASI(+)RyR1). RyR1-knockout myotubes expressing ASI(-) exhibited a decreased incidence of Ca^2+^ oscillations^19^ and a homozygous mutation at an exon (P3527S, exon71) adjacent to that of ASI has been identified in central core disease (CCD)^20^. These studies above indicate that alternative splicing of ASI plays an important role in the regulation of RyR1 when adapted to different physiological and pathological conditions. In the present study we found that the fetal variant ASI(-) isoform was significantly increased in atrophic skeletal muscles which was regulated by the up-regulation of CUG-BP1. Thus, our findings provide new insight into the regulation of alternative splicing of RyR1 by CUG-BP1 in muscle diseases.

Altered myoplasmic Ca^2+^ handling plays an important role in muscle atrophy and myotonic dystrophy^22^,^34^,^43^,^44^. The aberrant Ca^2+^ signaling in muscle atrophy attributes to the activation of enzymes that involved in protein breakdown such as calpain^45^ and defective SR Ca^2+^ release has also been reported in age-dependent muscle weakness^46^,^47^. Alternative splicing, which is an important contributor for the distortion of calcium channel gene activity, significantly alters the Ca^2+^ handling in myocytes^21^,^48^. Intriguingly, recent studies demonstrated that calcium also impacts on the output of gene expression through altering alternative pre-mRNA splicing patterns^49^. Previous studies on DM1 showed that CUG-BP1 participates in the regulation of alternative splicing of calcium channels, such as exon 29 of Ca_v_1.1 and C-terminal of SERCA1b^19^,^48^. Here, we found that CUG-BP1 directly regulated alternative splicing of ASI isoform of RyR1. However, more detailed studies need to be performed over the link between impaired Ca^2+^ release and alternative splicing regulation of calcium channel genes by CUG-BP1 in muscle atrophy.

## Methods

### Animal model

The use of animals and experimental procedures were approved by the Institutional Animal Use and Care Committee of Institute of Biophysics at Chinese Academy of Sciences. The experiments were carried out in accordance with the approved guidelines. C57BL/6 male mice (age 8 weeks, weight about 21 g) were purchased from Weitong-Lihua Co. Denervation, disused and starvation-induced muscle atrophy models were made as previously described^50^. Muscle samples were harvested at 3, 7, 10 and 14 days after the denervation surgery.

### Patient samples and controls

The local Institutional Review Board of the Xijing Hospital approved the studies in human muscle tissue samples (KY20150318-2) and informed consent was obtained from all subjects. Human Gastrocnemius muscle samples were obtained from patients undergoing neurogenic atrophy who were previously diagnosed in strict accordance with the approval guidelines and regulations from the Xijing Hospital. All tissue samples were obtained from anonymous individuals without identifiers or demographic characteristics. The specimens were processed immediately after surgery and stored at liquid nitrogen until total RNA and protein were extracted.

### Plasmids

The DsRed tagged mammalian expression plasmid for CUG-BP1 was generated by PCR-mediated cloning based on the human CUG-BP1 expression vector (a gift from Dr. Hua Lou’s lab, Case Western Reserve University). The PCR products of Dsred coding sequence were digested with BamH I and Bgl II, and cloned into the hCUG-BP1 expression vector digested with BamH I.

For knockdown of CUG-BP1, two shRNA constructs were designed by BLOCK-IT™ RNAi Designer (http://rnaidesigner.lifetechnologies.com/rnaiexpress/) (shRNA-1: GCTCAAACGTTCGAAGAA, shRNA-2: GAGCCAACCTGTTCATCTA). The oligos that synthesized by Sangon Co. were annealing and cloned into pSIREN-RetroQ vector (Clontech). An oligonucleotide that target eGFP (5′-AAAGGACGGAGGACATTAT-3′) was cloned into pSIREN-RetroQ vector as control. The independently expressed Dsred fluorescent protein in pSIREN-RetroQ vector allows us to directly monitor the delivery.

### RNA Immunoprecipitation

Mouse C2C12 myoblasts were maintained in H-DMEM (Gibco) supplemented with 15% fetal serum (FS) and antibiotics, at 37 °C and 5% CO_2_. RIP experiment was performed as previously described^51^. Briefly, antibody to CUG-BP1 (Santa Cruz Biotechnology, cat# sc-20003), and IgG (Santa Cruz Biotechnology, cat# sc-2025) was added to supernatant for immunoprecipitation (IP) and mock group, respectively. Twenty microliters of protein A/G beads (Santa Cruz Biotechnology, cat# sc-2003) were added in the supernatant following the adding of antibody and IgG. After washing, ten percent of the beads were used for western blot analysis of pull-down CUG-BP1, the others were resuspended in 1 ml of Trizol to isolate coprecipitated RNAs according to the manufacturer’s instruction (Invitrogen).

### Quantitative PCR and RT-PCR analysis

One microgram of RNA were converted to first strand cDNA using PrimeScript^TM^ RT reagent kit with gDNA Eraser (Taraka, cat.#RR047A) following the manufacturer’s instruction. The quantitative PCR was carried out with a Rotor-Gene Q machine (Qiagen, Hilden, Germany) using a three-step protocol and PCR reaction for each sample was performed in triplicate. Data analysis was performed as previously described^50^.

Endogenous RyR1 ASI alternative splicing in muscle was analyzed with specific primers for mouse and human. The PCR product was separated on an 8% polyacrylamide gel in TBE buffer. Quantification of RyR1 ASI inclusion was determined by NIH Image J software, the percentage of RyR1 ASI(+) proportion was calculated as [ASI(+)/(ASI(-)+ASI(+)] × 100%^50^. Primers used in quantitative PCR and RT-PCR are shown in table 1.

### Electroporation of FDB muscle

*In vivo* transfection experiments were carried out on 8-week-old C57BL/6 mice according to a published procedure^33^. Briefly, Mice were first anaesthetized and received a single injection of 0.4 U of hyaluronidase (Sigma) in 25 μl PBS into the FDB muscle. After 1 h, a total of 10μg of plasmid DNA was injected into each FDB. An electrical field was then applied to the muscle to ensure the absorption of the plasmid. Electroporate the muscles by applying 20 pulses, 20 ms in duration/each with an electric field of 100 V/cm. None persistent injury and inflammation response of the muscles were observed 5 days post plasmids injection. Ten days after electroporation, mice were euthanized and muscles were collected.

### FDB single fiber preparation and Ca^2+^ measurement

FDB fibers were isolated by enzymatic disassociation in a Tyrode solution containing 100 μM Ca^2+^, 2 mg/ml type II collagenase (Sigma-Aldrich), 10mg/ml BDM for 60 min at 37 °C twice and loaded with 10 μM Fluo-4-AM (Molecular Probes, Eugene, OR, USA) for 15 min at room temperature. Mean FDB fiber size was 1 mm × 20 μM.

For determination of cytoplasmic Ca^2+^ transients and total SR Ca^2+^ store, individual FDB fibers were transferred to an experimental chamber at room temperature in Tyrode solution containing (in mM) 140 NaCl, 5.5 KCl, 2 CaCl_2_, 1 MgCl_2_, 10 HEPES, 3 glucose, pH 7.4. Ca^2+^ was measured by using a laser scanning confocal microscope (SP5, Leica), which was connected to Leica DMI6000 inverted microscope, using a Plan Apo × 40 oil objective. The myocytes were first excited at 543nm to detect the DsRed expression. Then the excitation wavelength was changed to 488nm to detect the Ca^2+^ fluorescence activity of the DsRed positive cells. Fibers were imaged in full-frame (XY) mode, with a format of 512 × 512 pixels, a total time lapse of 250 images were acquired at 1 Hz. Images were processed and analyzed using Image J. The Ca^2+^-dependent fluorescence intensity ratio (F/F0) was plotted as a function of time. Under our experimental conditions, fluorescent bleaching was not significant.

### Western blot analysis

Tissues from control and atrophy skeletal muscle was isolated from anesthetized mcie and homogenized in RIPA buffer containing 50mM Tris (pH7.4), 150mM sodium chloride,1% Triton X-100, 1% sodium deoxycholate, 0.1% SDS. After centrifugation to remove cell debris, the lysates were prepared using loading buffer containing 2% SDS and 1% β-mercaptoethanol, pH8.8. Protein samples were heated at 85°C for 5min and a short spin every time before loaded onto a 12% SDS-PAGE in the running buffer. The resolved proteins were transferred to PVDF membranes (Millipore) at 300mA for 90 min. The membranes were blocked for 1h with Tris-buffered saline-Tween 20 (TBST) containing 1% BSA at room temperature. The blocked membranes were immunoblotted with specific antibodies. After washing 5 times in TBST, the membranes were incubated in TBST containing 1% BSA and the secondary antibody conjugated to horseradish peroxidase. Final detection was performed using enhanced chemiluminescence detection solution 1 and 2 (1:1) (ECL; Millipore). Relative expression level of protein was analyzed by densitometry using Image J program (Bethesda, Maryland, USA). Primary antibodies used in this study were as follows: anti-CUG-BP1 (Eptomics), Anti-GAPDH (Invitrogen, USA), anti-Histone 3 (Cell Signaling, USA) antibody). β-actin (Beyotime, China) protein was detected as a control housekeeping protein to ensure equal protein loading in all experiments.

### Immunostaining

The slides with muscle sections were fixed with 3% paraformaldehyde for 20 min and washed with PBST (0.3% Triton X-100 in PBS) for 10 min and repeat three times. The slides were incubated with anti-monoclonal CUG-BP1 antibody overnight at 4 °C. Then, the slides were washed with PBST for three times and cultured with goat anti-mouse secondary antibody (Alexa Fluor® 546, Invitrogen) for 2 h. Slides were washed with PBST for three times and following by staining with DAPI (Vector Laboratories) for 10 min and wash with PBST. The slides were examined with a fluorescent microscope (Leica).

### Nuclear protein extraction

To prepare the nuclear proteins, TA or Gas muscles were lysed using tissue homogenizer and a nuclear and cytoplasmic protein extraction kit (Beyotime, China) according to the manufacturer’s instructions. The lysates were ultracentrifuged at 12,000 × g for 10 min at 4 °C, and the supernatants were collected as the cytoplasmic fraction. The pelleted nuclei were resuspended in a buffer containing 1 mM PMSF. After 30 min at 4 °C, lysates were centrifuged, and supernatants containing the nuclear proteins were stored at −80 °C.

## Additional Information

**How to cite this article**: Tang, Y. *et al.* CUG-BP1 regulates RyR1 ASI alternative splicing in skeletal muscle atrophy. *Sci. Rep.* **5**, 16083; doi: 10.1038/srep16083 (2015).

## Supplementary Material

Supplementary Information

## Figures and Tables

**Table 1:** List of primers used in this study.

Name	Sequence (5′-3′)
Human-RT-ASI-F	GTCCAGAATGAGATCAACAACATGTC
Human-RT-ASI-R	CCGGCGCTTCTTCTTGGTGCGTTCCTG
Mouse-RT-ASI-F	GTCCAGAATGAAATCAACAACATGTC
Mouse-RT-ASI-R	CCGGCGCTTCTTCTTGGTGCGTTCCTG
Mouse-ASI(+)-qPCR-F	CTTTAAGCGTGAGAACAGAAC
Mouse-ASI(+)-qPCR-R	CCACCTGACTGTACGTCTCCTG
CUG-BP1-RIP-RyR1-F	CACAACAGCCCTCTCTGGGGTTC
CUG-BP1-RIP-RyR1-R	CGCTGACCTGTACGTCTCCTG
Atrogin-1-F	TGAATAATCCCAGCACACGA
Atrogin-1-R	ATCGGCAACTGCATCTCTTC
MuRF-1-F	TAACTGCATCTCCATGCTGGTG
MuRF-1-R	GGCGTAGAGGGTGTCAAACTT
LC3-F	CGGCTTCCTGTACATGGTTT
LC3-R	ATGTGGGTGCCTACGTTCTC
Bnip3-F	CACCTTCTGATGAAGATTTGGA
Bnip3-R	GGAACACCGCATTTACAGAA
