# Supplementary material for: CUG-BP1 regulates RyR1 ASI alternative splicing in skeletal muscle atrophy
Source: Sci Rep. 2015 Nov 4;5:16083. doi: 10.1038/srep16083 (PMC4632035; doi:10.1038/srep16083)
Supplement: Supplementary Information [file srep16083-s1.pdf]

CUG-BP1 regulates RyR1 ASI alternative splicing in skeletal muscle atrophy

Yinglong Tang[1,3,#], Huiwen Wang[1,#,*], Bin Wei[2,#], Lei Gu[1,3], Zhiguang Yang[1,3], Yuting Guo[3], Qing Zhang[5], Yanyun Wu[1], Qi Yuan[1], Gang Zhao[4,*] and Guangju Ji[1,*]

[1]National Laboratory of Biomacromolecules, Institute of Biophysics, Chinese Academy of Sciences, 15 Datun Road, Beijing 100101, China

[2]Department of Physiology, Wayne State University School of Medicine, Detroit, Michigan 48201

[3]University of the Chinese Academy of Sciences, Beijing 100049, China

[4]Department of Neurology, Xijing Hospital, Forth Military Medical University, Xi'an, Shaanxi 710032, China

[5]Department of Anesthesiology, Beijing Tiantan Hospital, Capital Medical University, Beijing 100050, China

#Contributed equally to this work.

*To whom correspondence should be addressed to: Guangju Ji (gj28@ibp.ac.cn), Huiwen Wang (whw@moon.ibp.ac.cn), or Gang Zhao (zhaogang@fmmu.edu.cn)

Supplementary figures

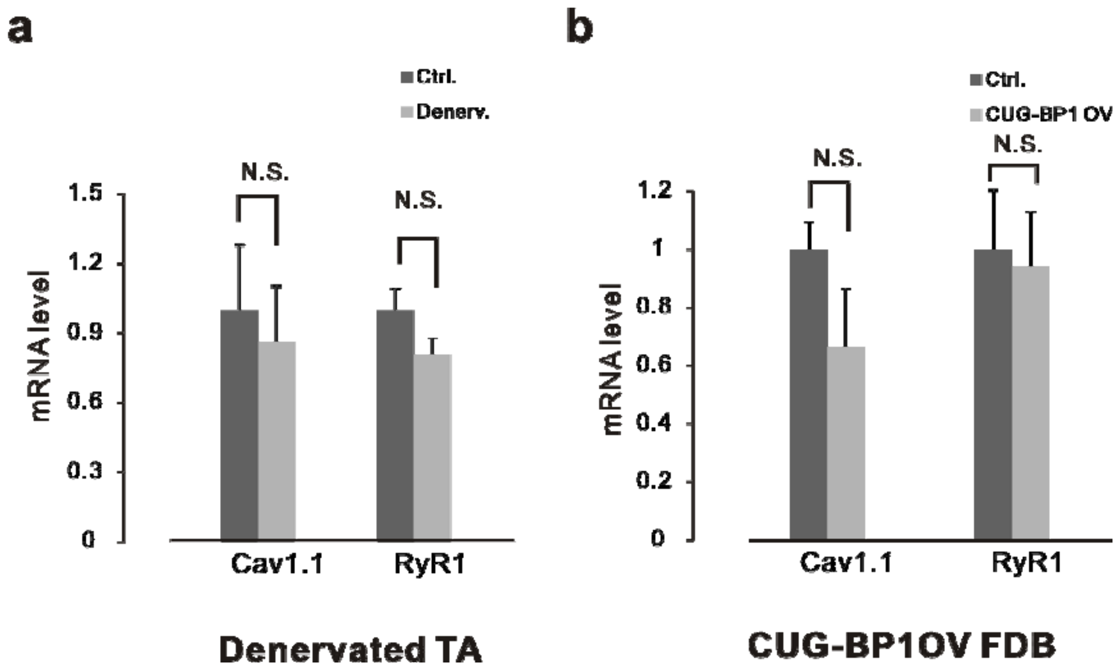

Figure S1. The mRNA expression level of Ca^{2+} release elements in denervation and CUG-BP1 overexpressed FDB muscle.

Description: The mRNA level of RyR1 and Cav 1.1 were not changed both in the denervation **(a)** and CUG-BP1 overexpressed **(b)** FDB muscle by real-time PCR assay.

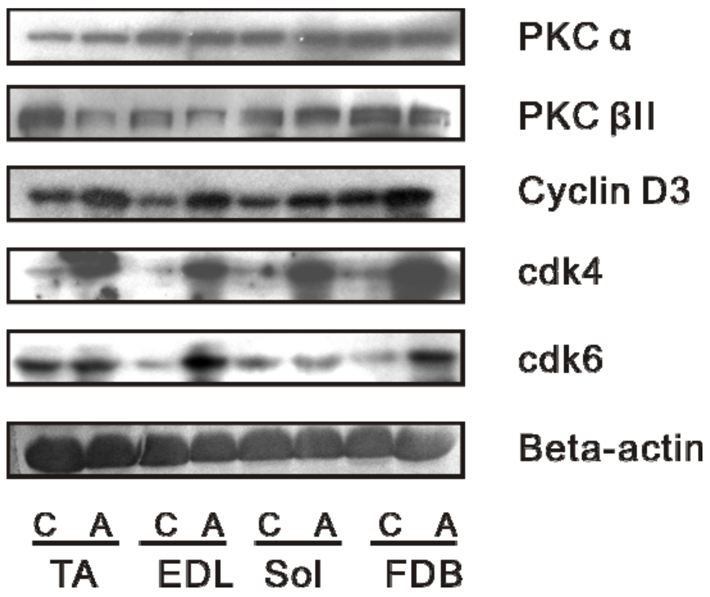

Figure S2. Cyclin D3/cdk4/6 protein level is increased in denervation-induced muscle atrophy but not PKC.

Description: representative blots show Cyclin D3 and cdk4/6 protein levels were significantly increased in TA, EDL, soleus and FDB muscles seven days after denervation. Neither PKC α nor PKC βII protein level was statistically altered in muscles after denervation. All results were repeated for three times.
